# Feasibility of FDCT Early Brain Parenchymal Blood Volume Maps in Predicting Short-Term Prognosis in Patients With Aneurysmal Subarachnoid Hemorrhage

**DOI:** 10.3389/fneur.2022.888369

**Published:** 2022-07-14

**Authors:** Lili Wen, Longjiang Zhou, Qi Wu, Xiaoming Zhou, Xin Zhang

**Affiliations:** ^1^Department of Neurosurgery, Sir Run Run Hospital, Nanjing Medical University, Nanjing, China; ^2^Medical Imaging Center, The Affiliated Hospital of Yangzhou University, Yangzhou, China; ^3^Department of Neurosurgery, Jinling Hospital, Nanjing University School of Medicine, Nanjing, China

**Keywords:** perfusion, parenchymal blood volume, digital subtraction angiography, outcome, subarachnoid hemorrhage

## Abstract

**Purpose:**

Aneurysmal subarachnoid hemorrhage (SAH) is accompanied by cerebral perfusion changes. We aimed to measure the parenchymal blood volume (PBV) maps acquired by C-arm flat-panel detector CT (FDCT) to assess the cerebral blood volume at an early stage in aneurysmal SAH and to explore the correlation with the outcomes at discharge.

**Methods:**

Data of 66 patients with aneurysmal SAH who underwent FDCT PBV examination were retrospectively analyzed. The PBV of regions of interest, including the cortices of the bilateral frontal lobe, the parietal lobe, the occipital lobe, and the cerebral hemisphere, as well as the basal ganglia, were measured and quantitatively analyzed. The clinical and imaging data of the patients were also collected, and logistic regression analysis was performed to explore the correlation between the perfusion parameters and outcomes at discharge.

**Results:**

The favorable and poor outcomes at discharge were found in 37 (56.06%) and 29 (43.94%) patients, respectively. The whole-brain PBV was significantly correlated with the Hunt-Hess grades (*p* < 0.005) and the WFNSS grades (*p* < 0.005). The whole-brain PBV of the poor prognosis was significantly higher than that of the favorable prognosis (35.17 ± 7.66 vs. 29.78 ± 5.54, *p* < 0.005). The logistic regression analysis showed that the PBV of the parietal lobe at the bleeding side (OR = 1.10, 95%CI: 1.00–1.20, *p* = 0.04) was an independent risk factor predicting the short-term prognosis.

**Conclusions:**

Parenchymal blood volume (PBV) maps could reflect the cerebral blood volume throughout the brain to characterize its perfusion status at an early stage in aneurysmal SAH. It enables a one-stop imaging evaluation and treatment in the same angio-suite and may serve as a reliable technique in clinical assessment of aneurysmal SAH.

## Introduction

Aneurysmal SAH is a life-threatening disease with high mortality and disability rates (1–4). Increasing fundamental and clinical research has suggested that early brain injury (EBI) is the most critical cause of the subsequent delayed cerebral vasospasm, delayed neurological dysfunction, and mortality and disability in patients (5–10). EBI, which involves a series of microcirculation dysfunctions that occur within 72 h after SAH, was correlated with early cerebral hypoperfusion, which was responsible for the subsequent delayed cerebral infarct (DCI) and poor prognosis of patients (11, 12).

Several investigators have found that about 60% of patients showed abnormal perfusion on MR perfusion (MRP) at an early stage of aneurysmal SAH, which was correlated with the Hunt-Hess grade and neurological prognosis (13, 14). Also, several studies reported that, after aneurysmal SAH, CT perfusion (CTP) can reflect the severity of brain injury and predict the occurrence of delayed cerebral ischemia (DCI) (15–22), in which decreased CBF and prolonged mean transit time (MTT) in the early stage of aneurysmal SAH were found to be related to the DCI and the poor outcome (18, 21). Given the fact that the reversal of vasospasm does not appear to improve patient outcomes, it could be argued that the earlier diagnosis and treatment of EBI may attenuate some of the devastating secondary injuries and improve the outcome of patients with SAH (5).

However, both MRP and CTP involve a relatively long waiting time before the examination and require the patients to be transferred to the special examination room, which are both challenging for patients with aneurysmal SAH (especially high-grade patients) when they are in a serious condition and need emergent surgery. Therefore, clinical applications of MRP and CTP are still limited. An alternative to MRP or CTP that is easily accessible, effective, and accurate is of great importance in clinical practice.

The C-arm flat-panel detector CT (FDCT) *syngo* DynaPBV Neuro is a 3D imaging application that provides the cerebral blood volume parameter intraoperatively for perfusion status assessment developed with the advancement of computer and imaging technology in recent years (23–28). The application visualizes the contrast-enhanced blood volume distribution of the whole brain in 3D color-coded cross-sectional images based on a steady-state contrast injection. It also allows measurements of PBV to quantitatively assess the perfusion changes caused by treatment or the biological processes. The PBV measurement can be performed in the same angio suite together with the interventional surgery in a one-stop fashion, which is safe and convenient for patients. Several authors reported that the cerebral blood volume calculated by PBV software compared favorably with that measured with CTP, and PBV's ability in cerebral perfusion evaluations is similar to CTP (26, 29). PBV has been found useful in evaluating perfusion in patients with acute ischemic stroke (AIS) (24–26, 28), yet its application in aneurysmal SAH is still in its infancy (23, 30).

In this study, we used the C-arm FDCT *syngo* DynaPBV Neuro application to measure the cerebral PBV and evaluate the association between the cerebral perfusion status at an early stage in aneurysmal SAH and the clinical manifestations in patients, as well as the functional outcomes at discharge. We hypothesized that PBV would serve as a reliable technique for the evaluation of hemorrhage severity and prediction of short-term prognosis in patients with aneurysmal SAH.

## Materials and Methods

### Patients

The study was approved by the institutional research ethics committee of Jinling Hospital, Nanjing University, Nanjing, China. Written informed consent was obtained from a legally authorized representative of all patients. Data of patients diagnosed with aneurysmal SAH who underwent C-arm FDCT PBV examination in the early stage (<48 h) in the Jinling Hospital between 1 January 2016 and 31 December 2018 were retrospectively analyzed. The exclusion criteria were the following: DSA or CTA suggested the presence of intracranial hematoma with local mass effect, cerebrovascular malformation, moyamoya disease, moderate or higher degree cerebral artery stenosis, or other cerebrovascular diseases and patients who had already received external ventricular drain. Clinical records related to functional outcomes including age, gender, Hunt-Hess grade, World Federation of Neurological Societies Scale (WFNSS) grade, modified Fisher (mFisher) grade, as well as the location of the aneurysm, were collected for analysis.

### Methods

All patients received a whole-brain perfusion examination on the C-arm FDCT (Artis Zee Biplane, Siemens Healthineers, Forchheim, Germany) through the transfemoral artery approach within 48 h after hemorrhage. C-arm FDCT PBV was acquired after general anesthesia in the angio-suite. As previously described (25, 31), PBV acquisition includes two 3D rotations: mask and fill runs. For both runs, the C-arm rotated 200° in 6 s, with an angle increment of 0.5°.

The first 3D mask run was acquired with no contrast filling. When the C-arm returned to the initial position after the mask run, 80 ml of 1:1 diluted contrast media (iodixanol, Visipaque 320 mg I/ml, GE Healthcare, Ireland) was injected through a 5F pigtail catheter placed at the aorta root at 8 ml/s, 600 psi for 10 s. To ensure the contrast filling in the brain tissue has reached the steady-state, the second 3D fill run was not triggered until superior sagittal sinus filling was observed during “bolus watching” (32).

**Figure 1:**
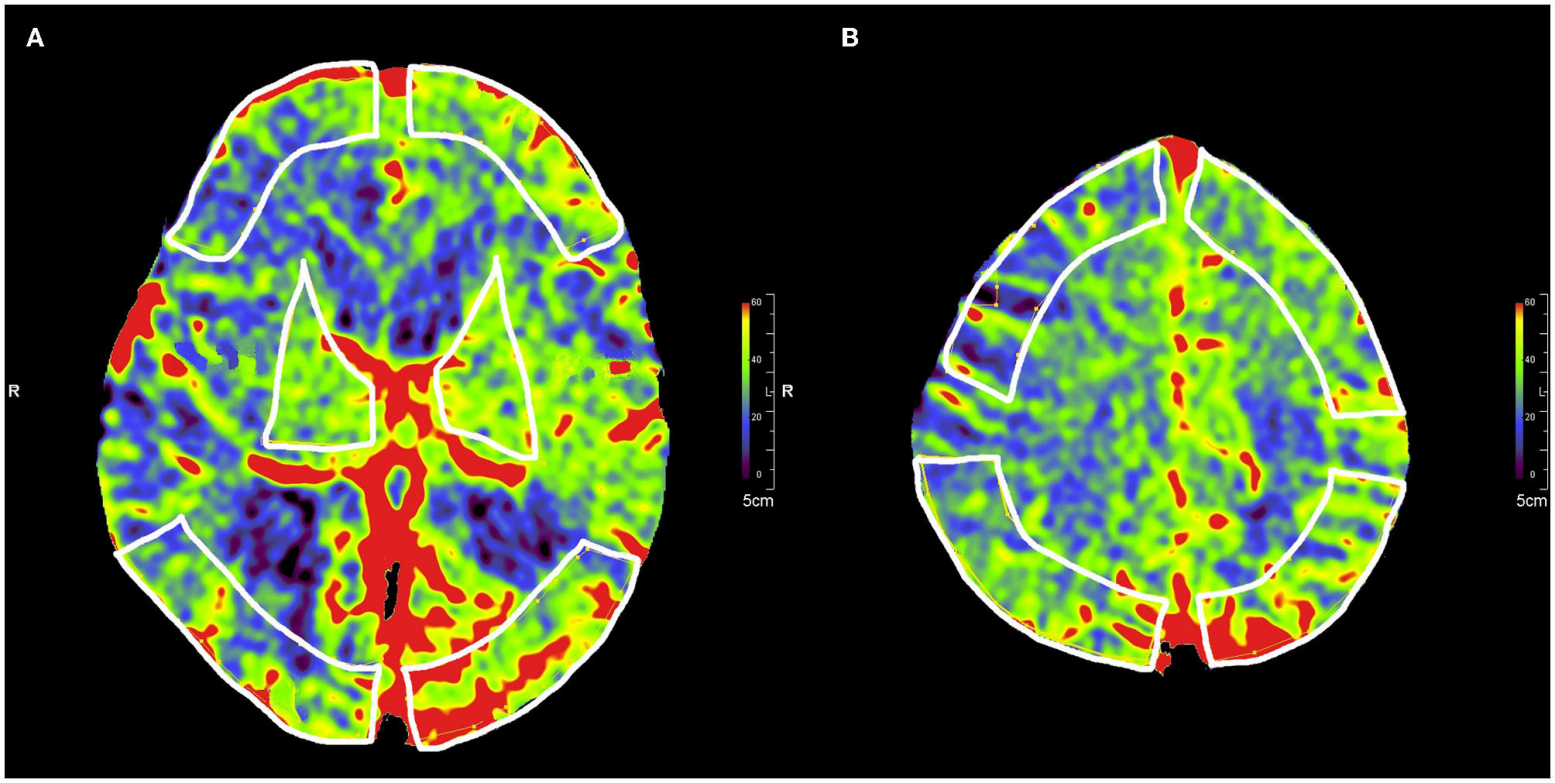
Selection of five symmetrical ROIs in color-coded parenchymal blood volume maps. **(A)** ROIs of the frontal pole cortex, the occipital cortex, and the basal ganglia in the third ventricle level. **(B)** ROIs of the frontal cortex and the parietal cortex at 4.5 cm above the third ventricle level.

Post-processing of the 3D data to generate color-coded PBV maps was performed using the *syngo* DynaPBV Neuro software (Siemens Healthineers, Forchheim, Germany) on the clinical workstation (*syngo* X workplace, Siemens Healthineers, Forchheim, Germany). In brief, PBV map reconstruction includes a subtraction of the mask image (1st run) from the fill image (2nd run) and detection of the arterial input (33). The PBV values were measured in units of ml/1,000 ml of cerebral tissue and viewed with a thickness of 10 mm using MPR. Then, five symmetrical regions of interest (ROIs), excluding hematoma, were drawn on the perfusion maps on the bilateral cerebral hemispheres for each patient: (1) Third ventricle level: the bilateral frontal pole cortex, the occipital cortex, and the basal ganglia (Figure 1A); (2) 4.5 cm above third ventricle level: the bilateral frontal cortex and the parietal cortex (Figure 1B).

PBV_hemisphere_ = 0.2^*^(PBV_fontal pole_ + PBV_frontal lobe_ + PBV_parietal lobe_ + PBV_occipital lobe_ + PBV_Basal ganglia_), PBV_whole brain_ = 0.5^*^(PBV_left hemisphere_+ PBV_right hemisphere_). The whole cerebrum was divided into the bleeding side hemisphere and the non-bleeding side hemisphere according to the location of the aneurysm. If the aneurysm was located in the basilar artery, the hemisphere with more hemorrhage was determined as the bleeding side hemisphere.

### Clinical Outcomes

The modified Rankin score (mRS) of the patients at discharge was used for the evaluation of functional outcomes. The mRS of 0–2 points indicated favorable outcomes and 3–6 points indicated poor outcomes.

### Statistical Analysis

Continuous variables in this study (PBV values) are presented as mean with standard deviation (SD). Comparisons between groups were performed with analysis of *t*-tests or the Mann-Whitney-U-test for continuous parameters and the χ2 test for categorical parameters. Significant univariate factors with a *p*-value ≤ 0.1 were entered into multivariable logistic regression (forward). Odds ratios (OR) and associated 95% confidence intervals (CI) are reported for regression analysis. Statistical analysis was performed using IBM SPSS Statistics software Version 19.0 (IBM, Armonk, New York, USA). A *p*-value < 0.05 was considered statistically significant.

## Results

A total of 66 patients (24 men [36.4%] and 42 women [63.6%] with the mean age of 55.98 ± 9.99 years) were included in this study. Fifty-five patients (83.33%) were diagnosed with an anterior circulation aneurysm and 11 patients (16.67%) with a posterior circulation aneurysm. Favorable outcomes were achieved in 37 patients (56.06%) at discharge, while 29 patients (43.94%) were discharged with poor outcomes (Table 1).

**Table 1:** Demographics and clinical features of patients with aSAH.

**Characteristics**	**All patients (*N* = 66)**	**Favorable outcome (*n* = 37)**	**Poor outcome (*n* = 29)**	***P*-value**
Age (years), mean ± SD	55.98 ± 9.99	52.95 ± 9.33	59.85 ± 9.58	<0.005
**Gender**, ***n*** **(%)**				0.61
Men	24 (36.36%)	12 (32.43%)	12 (41.38%)	
Women	42 (63.64)	25 (67.57%)	17 (58.62%)	
**Hunt-Hess**, ***n*** **(%)**				<0.005
I	1 (1.5%)	1 (2.70%)	0 (0%)	
II	25 (37.9%)	22 (59.46%)	3 (10.34%)	
III	11 (16.7%)	7 (18.92%)	4 (13.79%)	
IV	20 (30.3%)	4 (10.81%)	16 (55.17%)	
V	9 (13.6%)	3 (8.10%)	6 (20.67%)	
**WFNSS**, ***n*** **(%)**				<0.005
I	25 (37.9%)	23 (62.16%)	2 (6.90%)	
II	9 (13.6%)	5 (13.51%)	4 (13.79%)	
III	3 (94.5%)	2 (5.41%)	1 (3.45%)	
IV	12 (18.2%)	2 (5.41%)	10 (34.48%)	
V	17 (25.8%)	5 (13.51%)	12 (41.38%)	
**mFisher**, ***n*** **(%)**				0.25
0	3 (4.5%)	3 (8.11%)	0 (0%)	
I	2 (3.0%)	2 (5.41%)	0 (0%)	
II	13 (19.7%)	11 (29.73%)	2 (6.90%)	
III	18 (27.3%)	8 (21.62%)	10 (34.48%)	
IV	30 (45.5%)	13 (35.14%)	17 (58.62%)	
**Aneurysm site**, ***n*** **(%)**				0.48
**Anterior circulation**				
ICA	22 (33.3%)	14 (37.84%)	8 (27.59%)	
ACA	3 (4.5%)	1 (2.70%)	2 (6.90%)	
AcomA	25 (37.9%)	12 (32.43%)	13 (44.83%)	
MCA	5 (7.6%)	3 (8.11%)	2 (6.90%)	
**Posterior circulation**				
PCA	2 (3.0%)	1 (2.70%)	1 (3.45%)	
BA	4 (6.1%)	4 (10.81%)	0 (0%)	
VA	5 (7.6%)	2 (5.41%)	3 (10.34%)	

*WFNSS, World Federation of Neurological Societies Scale; mFisher, modified Fisher; ICA, internal carotid artery; ACA, anterior cerebral artery; AcomA, anterior communicating artery; MCA, middle cerebral artery; PCA, posterior cerebral artery; BA, basilar artery; VA, vertebral artery*.

Blood volumes of the parietal lobe and the cerebral hemisphere on the bleeding side were significantly higher than those on the non-bleeding side, with 35.14 ± 9.71 vs. 33.17 ± 94, *p* = 0.01 for the parietal lobe and 32.59 ± 7.36 vs. 31.70 ± 04, *p* = 0.02 for the cerebral hemisphere. Although blood volume at the frontal lobe, the occipital lobe, and the basal ganglia on the bleeding side was higher than those on the non-bleeding side, statistical significance was not found (Table 2).

**Table 2:** Comparison of PBV on the bleeding side hemisphere and the non-bleeding side hemisphere.

	PBV^*^on bleeding side, ($\bar{x}\;\pm$SD)	PBV^*^on non-bleeding side, ($\bar{x}\;\pm$SD)	***P*-value**
Frontal lobe	30.70 ± 8.13	30.68 ± 6.89	0.98
Parietal lobe	35.14 ± 9.71	33.17 ± 8.94	0.01
Occipital lobe	34.12 ± 7.92	32.82 ± 8.53	0.16
Basal ganglia	30.42 ± 7.67	30.08 ± 7.85	0.54
Cerebral hemisphere	32.59 ± 7.36	31.70 ± 7.04	0.02

* *, PBV values were expressed in units of ml/1,000 ml*.

*PBV, parenchymal blood volume*.

Whole-brain PBV values increased significantly as the Hunt-Hess grades (*p* < 0.005) and the WFNSS grades (*p* < 0.005) increased. The whole-brain PBV of the poor outcome group was significantly higher than that of the favorable outcome group (35.17 ± 7.66 vs. 29.78 ± 5.54, *p* < 0.005) (Table 3).

**Table 3:** Comparison of whole-brain PBV with clinical features and outcomes.

	Whole brain PBV ^*^($\bar{x}\pm$SD)	***P*-value**
**Hunt-Hess**		<0.005
I-II	28.07 ± 5.33	
III	33.74 ± 5.07	
IV-V	35.20 ± 7.37	
**WFNSS**		<0.005
I-II	28.07 ± 5.33	
III	33.74 ± 5.07	
IV-V	35.20 ± 7.37	
**mFisher**		0.60
0	28.25 ± 7.49	
I	29.31 ± 2.39	
II	30.69 ± 5.19	
III	33.82 ± 6.82	
IV	32.35 ± 7.98	
**Aneurysm site**		0.82
Anterior circulation	32.24 ± 7.25	
Posterior circulation	31.69 ± 6.12	
**Outcome**		<0.005
Favorable	29.78 ± 5.54	
Poor	35.17 ± 7.66	

* * PBV values were expressed in units of ml/1,000 ml*.

*WFNSS, World Federation of Neurological Societies Scale; mFisher, modified Fisher. PBV, parenchymal blood volume*.

Multivariate logistic regression results showed that only the blood volume of the parietal lobe on the bleeding side resulted as the independent risk factor predicting the functional outcome in patients at discharge (OR = 1.10, 95%CI: 1.00–1.20, *p* = 0.04) (Table 4).

**Table 4:** Multivariate logistic regression analysis (forward) of factors associated with outcome at discharge.

**Characteristics**	**Mono-variate regression**		**Multi-variate regression**	
	**OR (95% CI)**	***P*-value**	**OR (95% CI)**	***P*-value**
Age	1.08 (1.02–1.14)	0.01	1.11 (1.03–1.19)	0.01
Hunt-Hess	3.33 (1.85–2.99)	0.00	−	0.74
WFNSS	2.27 (1.55–3.31)	0.00	2.17 (1.38–3.43)	<0.005
mFisher	2.44 (1.31–4.54)	0.005	−	0.13
Site	0.69 (0.18–2.61)	0.58	−	−
Bleeding side PBV_parietal lobe_	1.13 (1.05–1.22)	0.00	1.10 (1.00–1.20)	0.04
Bleeding side PBV_hemisphere_	1.15 (1.05–1.27)	0.00	−	0.97
PBV_whoe brain_		0.00	−	0.52

*WFNSS, World Federation of Neurological Societies Scale; mFisher, modified Fisher; PBV, parenchymal blood volume*.

## Discussion

As aforementioned, EBI plays a critical role in brain dysfunction, leading to the subsequent vasospasm, delayed neurological dysfunction, and mortality and disability. Research on early brain injury involves endothelial damage, changes in vascular smooth muscle contractility, vascular reactivity, and neuroinflammation, such as interleukin 6 (IL-6); a key component in the development of vasospasm, is related to the blood-brain barrier destruction. The aforementioned pathophysiological changes may lead to damage to the integrity of the neurovascular unit and result in impaired vascular autoregulation (34). To prevent further deterioration in patients with aneurysmal SAH, in addition to the general clinical evaluation, such as frequent neurological assessment and monitoring approaches, including cerebral microdialysis (CMD), cerebral EEG, and transcranial Doppler (TCD), to detect suspicious signs, prompt outcome prediction will better facilitate follow-up treatment and care delivery planning and balance medical resources to patients in greater need.

In brain imaging after aneurysmal SAH, MRP has high sensitivity in depicting brain abnormalities, which makes it a good candidate for identifying early signs of vasospasm and ischemia in patients with aneurysmal SAH; yet, it is less commonly used than CTP in clinical practice due to the technical difficulty and examination accessibility (35). CTP was found to be reliable in vasospasm and DCI prediction and detection after aneurysmal SAH, and the CBF and MTT obtained were analyzed and suggested to be diagnostic thresholds (36–39). The measurements of MTT and TTP obtained from early CT perfusion were also demonstrated to be correlated with early clinical outcomes (40). Neuro PBV maps obtained from CBCT are a technique to measure cerebral blood volume throughout the brain to characterize its perfusion status (23). The reliability of PBV maps was demonstrated by the good correlation between PBV and conventional CT perfusion through both qualitative and quantitative comparative studies (29, 33, 41). By extending the imaging capabilities of the angio-suite, the PBV technique has been used in the assessment of ischemic cerebrovascular diseases and brain tumors in the brain during the procedure in the angio-suite for better patient management, and has gained significant value in clinical practice in recent years (24–28, 42) but not much value in SAH yet. Our study demonstrated PBV's feasibility in assessing the perfusion status in aneurysmal SAH, as well as the convenience of one-stop imaging evaluation. The major finding of our study was that the cerebral blood volume given by PBV maps in the early stage of aneurysmal SAH was significantly correlated with the initial severity of hemorrhage and the short-term prognosis of patients, which may predict early clinical outcome and aid in treatment planning.

All patients in this study received a PBV examination within 48 h after hemorrhage, during which time the incidence of ultra-early vasospasm was low (43), to explore the relationship between PBV and severity of EBI. Cerebral angiography performed after the PBV examination excluded the patients who had acute cerebral vasospasm or moderate to severe stenosis. Both the FDCT-derived PBV and conventional CTP are acquired based on the bolus detection of contrast agent under x-ray; it is challenging to distinguish between the contrast extravasation and the hematoma (44). Therefore, patients with a large local hematoma were excluded from our study. In addition, we selected ROIs that did not include hematoma to avoid large bias and only reflect the cerebral parenchymal blood volume. Our study showed that the blood volume of the selected areas on the bleeding side was higher than those on the non-bleeding side, especially in the parietal lobe and the cerebral hemisphere where significant differences were observed. These results are in concordance with the cerebral pathological changes after aneurysmal SAH. CBF could maintain stability in patients with intact autonomic regulation (45–47), but increased intracranial pressure (ICP) and decreased CBF after aneurysmal SAH lead to congestive changes in brain tissue manifested by dilated cerebral arterioles and increased CBV in patients with impaired autonomic regulation (11, 48, 49). The blood supply of the parietal ROIs that we selected was covered by the middle cerebral artery, which responded immediately after hemorrhage and manifested as cerebral congestion, resulting in increased PBV in the parietal ROIs. The association between CTP and aneurysmal SAH has been investigated previously, and our findings about PBV in this study show similar trends. PBV map and CTP-CBV/MRP-CBV have good consistency in terms of both visual comparison of perfusion pseudo-color maps and the quantitative analysis of ROI (26, 29, 30, 50, 51). In addition to PBV's application in AIS evaluation (24–26, 28) and initial practice in the prediction of DCI after aneurysmal SAH (23, 30), our study broadens the clinical application of PBV in stroke management.

In this study, significant differences were found between the PBV of different Hunt-Hess grades and WFNSS grades, which implies that the more severe the brain tissue congestion in the early stage of aneurysmal SAH, the higher the Hunt-Hess grade and the WFNSS grade. The PBV of the poor outcome group was significantly higher than that of the favorable outcome group, and the logistic regression model revealed that the PBV was an independent risk factor that could predict a patient's short-term outcome. The Hunt-Hess grade, the WFNSS grade, and the mFisher grade are routinely used as the basis for patient triage and outcome predictors but are given based on subjective judgment. On the contrary, cerebral PBV is a numeric value obtained by standard procedures. Our results may indicate that PBV has the potential to act as an objective screening method to predict outcomes in aneurysmal SAH. Patients with elevated PBV may indicate impaired autonomic regulation of the brain and subsequent treatment (e.g., optimal- cerebral perfusion pressure (CPP) targeted therapy guided by intracranial pressure (ICP) monitoring) can be applied immediately to maintain a reasonable CPP and a stable CBF to avoid cerebral congestion or ischemia. Future studies with a larger patient population and long-term outcomes will be conducted to explore the diagnostic threshold for PBV so as to make more objective and accurate predictions, and guide clinical interventions in a timely manner (52). During the COVID-19 pandemic, not only was the coronavirus likely to worsen hypertension and make aneurysms more prone to rupture, but intensive care unit (ICU) resources were more strained, leaving patients more vulnerable (53). PBV may help in the future to early identify SAH patients with severe brain injury and a possible poor prognosis to better allocate medical resources to those most in need.

Compared with CTP and MRP, PBV has the following advantages: (1) PBV can be performed in the angio-suite when there is an endovascular surgery for ruptured aneurysms. The patients are exempted from additional waiting time. More importantly, a comprehensive analysis of cerebral perfusion and angiography could provide more clues for identifying abnormal cerebral perfusion caused by acute vasospasm and vascular abnormalities such as vascular stenosis; (2) The whole-brain volume reconstruction of PBV maps could visualize any slice of cerebral blood volume imaging on transversal, sagittal, and coronary views; (3) PBV realizes the imaging evaluation of patients with aneurysmal SAH together with other DSA techniques, such as 2-D angiography and color-coding blood flow analysis in the same angio-suite, which is the so-called one-stop imaging service in the angio-suite (54).

However, PBV only provides cerebral blood volume values at present but lacks perfusion parameters of CBF, MTT, and TTP. The evaluation of patients who have no obvious CBV abnormalities in the early stage, with only prolonged MTT and slightly decreased CBF, may be inaccurate (48). Improved PBV technology that can calculate more perfusion parameters in the future may help in a more comprehensive and accurate evaluation of the brain perfusion status (55). PBV acquisition currently still requires manual triggering in the fill run. The ideal time point for data collection is when the contrast agent reaches a steady-state filling in the brain capillary bed, that is, the concentration of the contrast agent in the artery = the concentration of the contrast agent in the tissue = the concentration of the contrast agent in the vein. However, manual triggering requires skilled operation and may result in inappropriate acquisition time, i.e., too early or too late when the contrast agent is not maintained in a steady-state equilibrium in the brain tissue. The PBV value component contains a part of CBF weight in this condition (23, 56, 57).

This study has several limitations: (1) This was a preliminary study with a relatively small sample size; (2) for ethical reasons, PBV examination was lacking in normal patients, which meant that the control group was not available; and (3) there was no discrimination between anterior and posterior circulation aneurysms. The accuracy of PBV measurement of the cerebellum and the brainstem may be affected due to the imaging limitations caused by the posterior fossa (58, 59). This study mainly focused on the feasibility of using PBV for cerebral perfusion status evaluation after aneurysmal subarachnoid hemorrhage. More systematic comparative studies of aneurysms in the anterior and posterior circulations need to be carried out.

## Conclusions

Our results demonstrated that the cerebral blood volume measured by PBV maps at an early stage in aneurysmal SAH is significantly correlated with the initial severity of hemorrhage and the short-term prognosis of patients. The C-arm FDCT PBV technique enables a one-stop imaging evaluation and may be a reliable alternative to CTP and MRP in clinical assessment and in predicting aneurysmal SAH outcomes.

## Data Availability Statement

The raw data supporting the conclusions of this article will be made available by the authors, without undue reservation.

## Ethics Statement

The studies involving human participants were reviewed and approved by the Institutional Research Ethics Committee of Nanjing Jinling Hospital. The patients/participants provided their written informed consent to participate in this study.

## Author Contributions

Study concepts and study design: LW and XZha. Data acquisition: LW, QW, and XZho. Quality control of data and algorithms and manuscript preparation: LW and LZ. Statistical analysis and interpretation: LW, LZ, and QW. Manuscript editing: LW. Manuscript review: XZha. All authors contributed to the drafting of this article.

## Funding

We declare that the present research was supported by Jiangsu Planned Projects for post-doctoral Research Funds (No. 2019k281), Jiangsu Natural Science Foundation (No. BK20191231) and Jiangsu Natural Science Foundation (No. SBK2019022915).

## Conflict of Interest

The authors declare that the research was conducted in the absence of any commercial or financial relationships that could be construed as a potential conflict of interest.

## Publisher's Note

All claims expressed in this article are solely those of the authors and do not necessarily represent those of their affiliated organizations, or those of the publisher, the editors and the reviewers. Any product that may be evaluated in this article, or claim that may be made by its manufacturer, is not guaranteed or endorsed by the publisher.

## Abbreviations

EBI: early brain injury
DCI: delayed cerebral infarct
PBV: parenchymal blood volume
FDCT: flat-panel detector CT
CBF: cerebral blood flow
MTT: mean transit time
AIS: acute ischemic stroke
WFNSS: world federation of neurological societies scale
mFisher: modified Fisher
mRS: modified rankin score.
